# Further evidence of the host plasticity of porcine circovirus-2: detection of the virus in domestic dogs in Namibia

**DOI:** 10.1186/s12917-025-04581-7

**Published:** 2025-03-12

**Authors:** Umberto Molini, Lourens de Villiers, Lauren M. Coetzee, Herschelle P. Green, Mari de Villiers, Siegfried Khaiseb, Giovanni Cattoli, William G. Dundon, Giovanni Franzo

**Affiliations:** 1 Istituto Zooprofilattico Sperimentale dell’Abruzzo e del Molise, 64100 Teramo, Italy; 2 Central Veterinary Laboratory (CVL), 24 Goethe Street, Private Bag 18137, Windhoek, Namibia; 3 School of Veterinary Medicine, Faculty of Health Sciences and Veterinary Medicine, University of Namibia, Neudamm Campus, Private Bag 13301, Windhoek, Namibia; 4 Faculty of Veterinary Medicine, University of Teramo, Teramo, Italy; 5 Istituto Zooprofilattico Sperimentale delle Venezie, 35020 Padua, Italy; 6 Animal Production and Health Laboratory, Animal Production and Health Section, Joint FAO/IAEA Division, Department of Nuclear Sciences and Applications, International Atomic Energy Agency, PO Box 100, Vienna, 1400 Austria; 7 Department of Animal Medicine, Production and Health, University of Padova, viale dell’Università 16, Legnaro, 35020 Italy

**Keywords:** PCV-2, Dog, Namibia, Molecular epidemiology, Host, Phylogenesis

## Abstract

Porcine circovirus 2 (PCV-2) is a major pathogen of swine, causing significant production losses in the pig industry worldwide. Several studies have detected the virus in different species, both in asymptomatic and diseased subjects, highlighting PCV-2 host plasticity. As PCV-2 has been identified in carnivores, the present study was undertaken to investigate the susceptibility of domestic dogs to PCV-2 infection by testing archived blood samples originating from low-income rural areas in Namibia. The population was selected considering the high exposure probability to PCV-2 due to direct contact and/or feeding with raw pig meat or by-products. Thirty-eight of the samples (6.61%) tested positive for PCV-2, and the complete ORF2 of 7 strains was sequenced, revealing the presence of the three major PCV-2 genotypes (i.e. PCV-2a, -2b, and -2d). Convincing epidemiological links with other Namibian and South African strains were established for PCV-2a and PCV-2b strains, while the PCV-2d strains were part of a broader clade that included sequences of viruses collected worldwide, especially in Asia. Although PCV-2 was reported in diseased dogs, no statistically significant or robust causal association between infection and disease manifestation was demonstrated. In conclusion, PCV-2 infection has been identified in Namibian dogs, most likely due to the ingestion of contaminated meat and by-products. However, the epidemiological and clinical consequences are still unclear and further investigations are necessary. Nevertheless, the high proportion of infected dogs showing clinical signs raises concern about the potential of PCV-2’s role as a relevant viral pathogen in Namibia. The use of raw meat for dog nutrition should be discouraged, considering the known risks to animal and human health through disease transmission.

## Introduction

Porcine circovirus 2 is a non-enveloped virus, member of the genus *Circovirus*, family *Circoviridae* and is characterized by a single-stranded circular DNA (ssDNA) genome of approximately 1.7 kb comprising 2 main open reading frames (ORFs) [1]. ORF1 encodes, through alternative splicing, the proteins Rep and Rep’, necessary for viral genome replication. ORF2 codes for the Cap protein, the only constituent of the viral capsid, which is involved in the attachment to the host cell via heparan sulfate (HS) and chondroitin sulfate B (CSB) glycosaminoglycans [2, 3]. The Cap is also the main target of the host immune response and, as such, is under strong selective pressures [4, 5]. Because of its high genetic variability, ORF2 is commonly used for molecular epidemiology studies and strain genotyping. PCV-2 [6], similar to other ssDNA viruses, is characterized by high mutation and recombination rates that have resulted in the emergence of variants, currently classified into 9 genotypes (i.e. PCV-2a-2i) based on the evaluation of ORF2 genetic distances and comparison with a set of reference sequences through phylogenetic analysis, as proposed by Franzo and Segales [6, 7]. Genotypes PCV-2a, −2b and −2d are considered the main genotypes due to their persistent, worldwide distribution, while the other six are reported more sporadically. In addition, among the main genotypes, a clear temporal pattern has been described. PCV-2a has been the predominant genotype until approximately 2003, after which PCV-2b (first genotype shift) predominated. In the following decade, around 2010, PCV-2d (second genotype shift) became predominant and currently represents the dominant genotype globally [7].

PCV-2 emerged as a devastating swine disease in the 90 s and was associated with several clinical syndromes, commonly named porcine circovirus diseases (PCVDs): PCV-2 systemic disease (PCV-2-SD), previously known as postweaning multisystemic wasting syndrome (PMWS) and comprising what was initially described as PCV2-associated pneumonia and PCV-2-associated enteritis, porcine dermatitis and nephropathy syndrome (PDNS); and PCV-2 reproductive disease (PCV-2-RD) [8]. PCVDs are typical examples of multifactorial diseases and PCV-2 infection does not imply disease manifestation, as most cases remain asymptomatic, i.e. PCV-2 subclinical infection (PCV-2-SI).

Despite being predominantly a swine infection, PCV-2 has remarkable host plasticity. The virus has been detected in wild boars, peccaries and warthogs [9–12] in addition to several non-Suidae species. These include bovines, rodents, antelope, and carnivores such as minks, foxes, raccoon dogs in China, and jackals in Namibia [11, 13]. However, when PCV-2 circulation was evaluated in Italy on a broad set of canine samples (described in [14, 15]), none were positive (Franzo, personal communication).

The explanation for the lack of PCV-2 infection in Italian dogs might lie in species-barriers preventing infection or alternatively, the absence of contact between domestic dogs and PCV-2-infected swine or pig by-products. Since contacts between dogs and swine in Italy are extremely rare, to explore this hypothesis further, an alternative scenario involving frequent contacts between dogs and pigs or their byproducts was considered. For these reasons, the current study involved biomolecular testing and sequencing of archived dog samples collected from low-income rural areas in Namibia, where feeding dogs with pig meat and by-products is common.

## Material and methods

### Sample collection

**Fig. 1:**
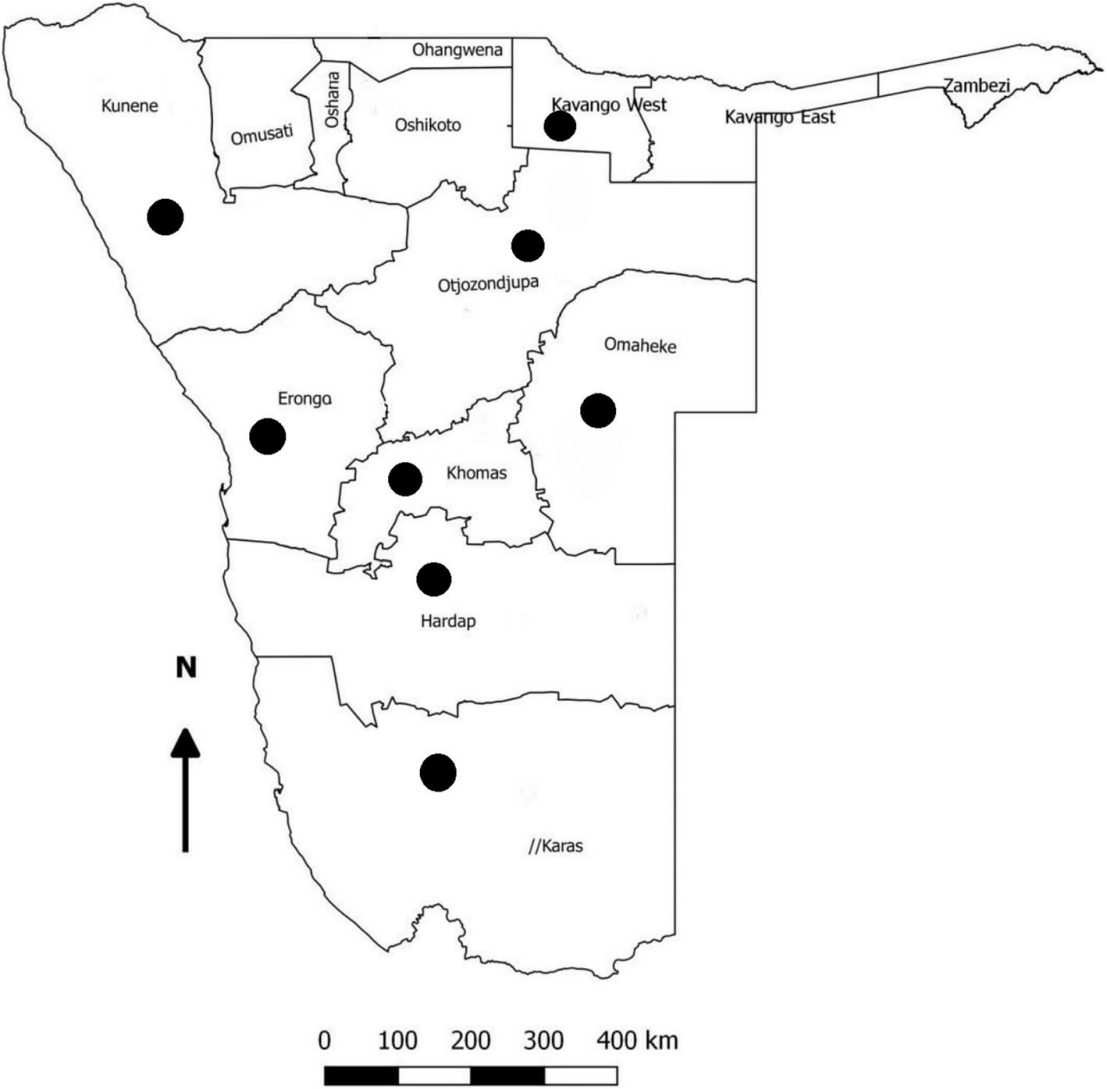
Map of Namibia indicating the regions from where the dog samples originated

Archived blood samples (n = 575) from domestic dogs were analyzed. The samples were collected between 2020–2022 from dogs presented to the Veterinary Academic Hospital for veterinary consultation (Table 1). The dogs originated from 8 different regions of Namibia, namely: Erongo, Hardap, Karas, Kavango-East, Khomas, Kunene, Omaheke, and Otjozondjupa (Fig. 1). Animal age, gender, and clinical signs were recorded and the association with PCV-2 infection was tested using a logistic regression model [16], setting the significance level at *p* < 0.05. Notably, the samples were previously also screened for the presence of canine circovirus (CanineCV), as reported by De Villiers et al., [17].

**Table 1:** Description of canine samples analyzed

Region	Town	Collection date	Number of dogs	PCV2-positive dogs		
				**n**	**Town %**	**Region %**
**Erongo**	Karibib	2022	24	1	4.17	6.98
	Usakos		19	2	10.53	
**Hardap**	Mariental	2022	40	4	10	7.35
	Rehoboth		28	1	3.57	
**Karas**	Keetmanshoop	2022	42	0	0	0
**Kavango-East**	Rundu	2022	59	7	11.86	11.86
**Khomas**	Windhoek	2020	19	0	0	3.70
		2021	58	2	3.45	
		2022	37	2	5.41	
	Groot Aub	2022	12	0	0	
	Brakwater		9	1	11.11	
**Kunene**	Khorixas	2022	47	5	10.64	13.70
	Outjo		26	5	19.23	
**Omaheke**	Witvlei	2022	57	1	1.75	2.41
	Gobabis		26	1	3.85	
**Otjozondjupa**	Grootfontein	2022	72	6	8.33	8.33
**Total**			**575**	**38**	**6.61**

### Extraction, PCR, and sequencing

Total genomic DNA was extracted from 200 µl of blood sample using the High Pure Viral Nucleic Acid Kit (Hoffman, Switzerland) with an elution volume of 100 μl, following the manufacturer’s instructions.

DNA extracts were screened using a real-time PCR (qPCR) assay with specific primers and probes for PCV-2, as reported by Franzo et al. 2020 [12]. Briefly, qPCR was performed on a C1000 Bio-Rad thermocycler (Bio-Rad Hercules, CA, USA) with PrecisionPLUS qPCR Mastermix (Genesig Primerdesign ltd, Camberley, UK). The cycling conditions were 95 °C for 7 min, followed by 45 cycles of 95 °C for 10 s, and 60 °C for 30 s. The fluorescence signal was acquired at the end of each cycle extension phase.

Two primers, PCV-ORF2-F1 (5'-TGCTACAGAACAATCCACGGA-3') and PCV-ORF2-R1 (5'- ACAGCGCACTTCTTTCGTTT-3'), producing an 853-bp segment were used to amplify the ORF2 of PCV-2 positive samples. The amplification performed with Taq DNA Polymerase (Thermo Fisher Scientific, Waltham, MA, USA) consisted of an initial denaturation at 95 °C for 5 min, followed by 35 cycles, each consisting of 95 °C for 30 s, 51 °C for 30 s, and 72 °C for 60 s, and a final elongation at 72 °C for 5 min.

Amplicons were purified using a Wizard SV Gel and PCR Clean-Up System (Promega) and Sanger sequencing was outsourced to the commercial company, LGC Genomics (Berlin, Germany). The sequences of the positive samples were submitted to the GenBank database under accession numbers OQ594429- OQ594435. The sequences were edited and assembled using the Staden software package version 2.0.0b8. Multiple sequence alignments were performed to compare and classify the PCV-2 sequences. Specifically, the full ORF2 reference dataset described by Franzo and Segalés (2018) [6] was downloaded and aligned to the ORF2 region of the sequences generated in this study using the MAFFT [18] method implemented in TranslatorX [19]. A maximum likelihood phylogenetic tree was performed using IQ-Tree [20], selecting as the best substitution model the one with the lowest Akaike information criterion, calculated using the same software. The robustness and reliability of the branching patterns were assessed by performing 10,000 ultrafast bootstrap replicates. Moreover, to further contextualize the detected strains within a PCV-2 molecular epidemiological framework, a complete ORF2 dataset of strains (n = 4902 sequences) was obtained from Franzo et al., [21]. Alignment and phylogenetic analysis were performed as described in the previous paragraph.

## Results

### Clinical signs and PCV-2 diagnosis

38/575 dog samples (6.61%) (Table 1), originating from all the regions involved in the study except for the Karas region, tested positive for PCV-2 by qPCR. The Cq values of the samples ranged between 29.59 and 35.88. The complete ORF2 genome was successfully sequenced in 7/38 positive dog samples (Table 2).

**Table 2:** Description of sequenced samples

ID	Region	Town	Strain	Ct value	N Genbank
50	Erongo	Usakos	PCV-2a	30.66	OQ594435
138	Kunene	Outjo	PCV-2a	29.84	OQ594429
248	Otjozondjupa	Grootfontein	PCV-2d	30.59	OQ594434
306	Kavango-East	Rundu	PCV-2d	32.50	OQ594432
528	Hardap	Mariental	PCV-2b	32.29	OQ594433
537	Hardap	Mariental	PCV-2b	32.22	OQ594431
665	Omaheke	Gobabis	PCV-2a	30.64	OQ594430

Overall, 329/575 (57.21%) of the dogs showed at least one of the following clinical signs: neurological, respiratory, enteric, or systemic (pyrexia, weight loss, and/or lymphadenomegaly). Among the diseased animals, twenty-five were PCV-2 positive, but no association was detected between PCV-2 infection and disease manifestation (*p* = 0.26). When clinical signs were evaluated individually, PCV-2 positive samples originated from dogs with neurological symptoms (*n* = 1), respiratory symptoms (*n* = 8), enteric symptoms (*n* = 0), pyrexia (*n* = 7), weight loss (*n* = 12), and lymphadenomegaly (*n* = 28), respectively.

Conversely, dogs with neurological symptoms (*n* = 4), respiratory symptoms (*n* = 68), enteric symptoms (*n* = 19), pyrexia (*n* = 84), weight loss (*n* = 96), and lymphadenomegaly (*n* = 295) were PCV-2 negative. A statistically significant association was detected between PCV-2 infection and weight loss (odds ratio = 2.30; 95 CI = 1.002—4.275; *p* = 0.039) and lymphadenomegaly (odds ratio = 2.306; 95 CI = 1.133- 5.078; *p* = 0.027).

After controlling for CanineCV infection status, no statistically significant association could be detected between PCV-2 infection and the presence of clinical signs, age, or gender. Notably, a significant association with CanineCV infection, tested in De Villiers et al. [17], as previously reported, suggests CanineCV-infected animals more likely to be PCV-2 positive (odds ratio = 3.2522; 95 CI = 1.669—6.3809; *p* < 0.001).

### Phylogentic analysis of PCV-2 strains

**Fig. 2:**
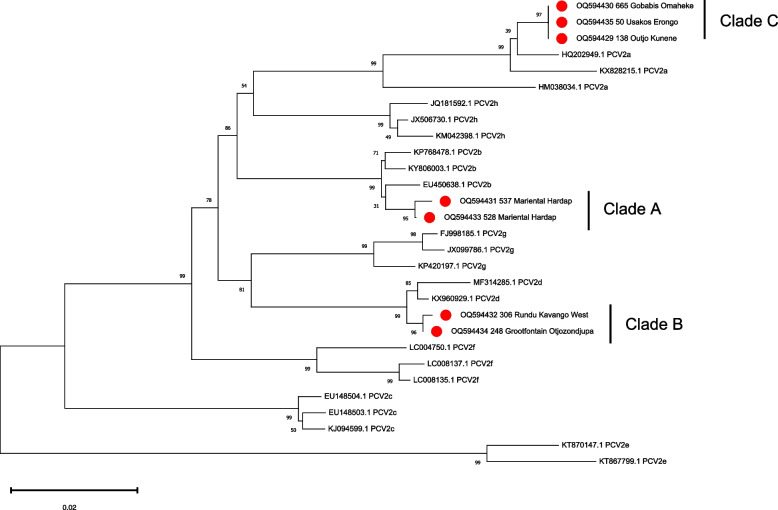
Maximum likelihood phylogenetic tree reconstructed with IQ-Tree based on PCV-2 ORF2 sequences obtained in the present study and reference sequences reported in Franzo & Segalés, 2018. The robustness and reliability of the branching patterns were assessed by performing 10,000 ultrafast bootstrap replicates. The clade name has been reported near the Namibian strains identified in the present study

**Fig. 3:**
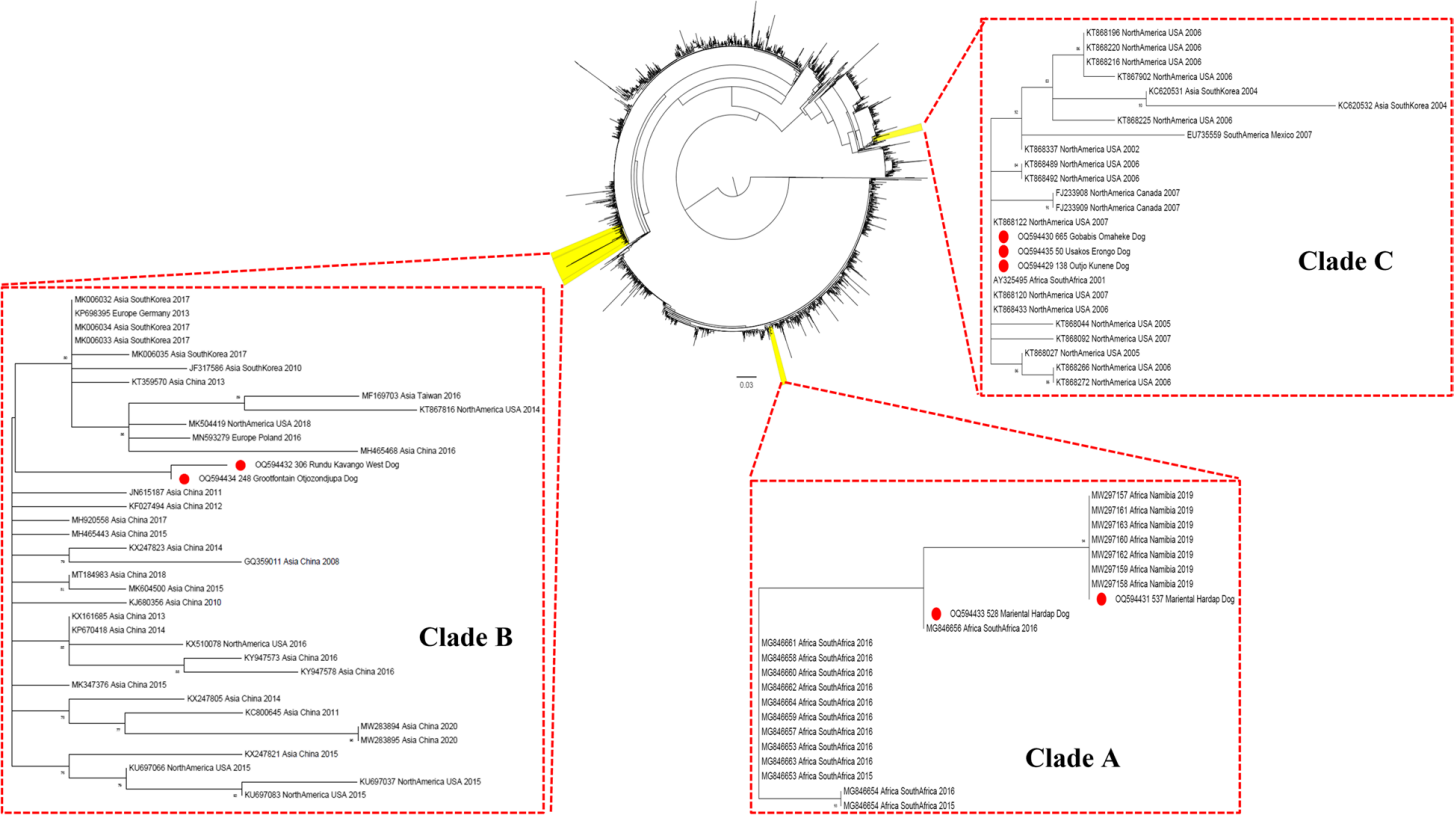
Maximum likelihood phylogenetic tree reconstructed with IQ-Tree based on PCV-2 ORF2 sequences. In the central figure, the clades of interest are highlighted in yellow. In the inserts, the sequences from Namibian dogs are marked with a red circle. The robustness and reliability of the branching patterns were assessed by performing 10,000 ultrafast bootstrap replicates

Comparison with the reference dataset of Franzo and Segalés (2018) showed that the sequences of the positive samples clustered with PCV-2a, PCV-2b, and PCV-2d genotypes (Fig. 2). When the sequences were compared with the larger, international dataset, they were classified into 3 different clades (herein renamed as Clade A, B, and C for the purposes of this manuscript only). Clade A (PCV-2b) comprised strains 537_Mariental_Hardap and 528_Mariental_Hardap and other sequences from Namibia and South Africa only. Strains 248_Grootfontein_Otjozondjupa and 306_Rundu_Kavango-East, although part of a distinct branch, were part of a much broader clade (Clade B; PCV-2d), including worldwide strains collected mainly in Asia, and also in Europe, North America, and a limited number (*n* = 2) in Africa (i.e. Mozambique and Zambia). Finally, PCV-2a strains (138_Outjo_Kunene, 50_Usakos_Erongo, and 665_Gobabis_Omaheke), part of Clade C, were closely related to North American and a single South African strain (Fig. 3). Analysis of the amino acid (aa) sequence of ORF2 in the PCV-2b strains 537_Mariental_Hardap and 528_Mariental_Hardap identified a longer capsid protein (i.e. 245aa rather than 233aa) of 12 amino acids (i.e. LMNNKNHYEVNE), due to an A → T mutation changing the stop codon sequence.

## Discussion

Although PCV-2 has previously been reported by Herbst and Willems in the feces of dogs the authors did not show that infection of the host had occurred [22]. Carnivore PCV-2 infections and dog susceptibility to PCV-3 have also been previously demonstrated [11], but this is the first report of PCV-2 infection in domestic dogs of Namibia. The high frequency of detection (> 6.6%) and the distribution of the virus throughout Namibia were unexpected.

Given the unexpectedness of the results, it was important that laboratory or sample contamination is ruled out. We are confident of the reliability of the results obtained for several reasons. The specificity of the real-time assay used for initial PCV-2 screening has been previously evaluated using a broad range of viruses, including other porcine and canine circoviruses. The samples that were confirmed by Sanger sequencing belonged to three different genotypes of PCV-2 and were not identical to each other. They were also different from the PCV-2a sample used as a positive control and from any of the other PCV-2 s identified previously in Namibia [10], thereby negating potential concerns of sample or laboratory contamination.

Considering the latter, the susceptibility of dogs to PCV-2 infection and the relatively high prevalence observed in Namibia can be claimed with confidence. The viral titers were low, as determined by the Cq values of the qPCR, explaining why only 7/38 samples were successfully sequenced. However, despite the low number of samples sequenced, three major PCV-2 genotypes were detected.

The two PCV-2b strains were part of a clade (Clade A) comprising only Namibian and South African strains, including the ones previously detected in Namibian jackals [13]. More specifically, the identified strains were closely related to ones previously reported in pig farms of the same town (i.e. Mariental) [10]. Mariental’s economical and employment associations to swine farming is relevant. Waste products (e.g. uncooked bones, offal, slaughtered pig meat, etc.) are commonly used for dog food and nutrition. Uncooked PCV-2 DNA-positive lymphoid tissues, bone marrow, and skeletal muscle from PCV-2 viremic pigs have previously been shown to be infectious for naïve pigs through oral consumption [23]. Therefore, ingestion of contaminated meat represents the most likely route of infection for the dogs tested. Direct dog-to-pig contact is an unlikely source of infection given the high biosecurity/containment practices employed by pig farmers in Mariental.

PCV-2 ORF2 proteins are normally 233 or 234 aa in length with an increase in virulence having been associated with the 234 aa length protein (Guo et al., 2012). Interestingly, the two PCV-2b strains from domestic dogs identified in this study had an extended capsid protein of 12 aa, because of a mutation in the stop codon. Differences in capsid length have already been reported in PCV-2 s with Davies et al. describing a 238 aa long ORF2 [24, 25]. This is the first instance where a 12 aa extension has been described. Whether this finding represents an adaptative change or results in a more pathogenic virus is currently unknown. Longitudinal monitoring of the canine population and the involvement of a broader population at a global level might assist in providing more clarity.

The PCV-2a sequences from Kunene, Erongo, and Omaheke were part of Clade C, consisting mostly of North American sequences and a single South African strain [26]. South Africa is one of the main exporters of both live swine and meat to Namibia, which might suggest an explanation to the presence of PCV-2a in Namibia, even though PCV-2a has never been identified in pigs in the country. It is therefore unclear whether the PCV-2a infection is confined to dogs or is circulating undetected in the pigs.

Finally, the two strains detected in Kavango-West and Otjozondjupa were classified as PCV-2d (Clade B) and were part of a homogeneous clade comprised mostly of Chinese strains. As PCV-2d has been previously identified in Namibian pigs [10, 21], the most likely explanation for detecting PCV-2d in dogs is ingestion of contaminated meat and/or by-products which originated from Namibian pig farms. However, given the strong economic ties between Namibia and Asian countries like China, and the frequent importation of pig meat, the source of PCV-2d infection might also be contaminated meat imported from other countries, which may have contributed to further PCV-2 spread in the dog population.

Something similar was proposed for peridomestic *M. musculu*s and *R. rattus* that were naturally infected with PCV-2 when captured on pig farms [11, 27]. Interestingly, contact with pigs appeared necessary, since rodents that were collected outside pig farms remained negative for PCV-2 [28]. By comparison, this could explain the lack of evidence of PCV-2 infection in Italian dogs, where dog contact with pigs is minimal and mainly commercial pet food, i.e. not pig meat or by-product, is used for dog nutrition. In contrast, it can therefore be speculated that the infection of PCV-2 in dogs in Namibia occurs following contact with either infected animals or their by-products.

The epidemiological and clinical consequences of PCV-2 infection of dogs are difficult to determine since this study was based on convenience sampling. Additionally, no archived sample materials were suitable for histological or immunohistochemistry analysis to determine viral antigen presence. Furthermore, no statistical evidence of an association between infection and disease manifestation was highlighted. Therefore, PCV-2 detection may represent an incidental findings in the presence of clinical signs ascribable to other diseases or infections. At the same time, the fact that several of the subjects showed overt clinical signs like lymphadenomegaly does not exclude a potential etiological role indisease emergence, at least as a contributing factor. Neurological signs were observed in one PCV-2 positive dog in this study, and previously in one CanineCV-positive dog [17]. Since CanineCV has been known to be associated with neurological disease in carnivores [29], whether PCV-2, CanineCV or co-infection were responsible for the clinical signs remains to be established. More generally, the increased risk of PCV-2 infection in CanineCV-positive dogs remain intriguing. Whether infection risk is ascribable to the exposure of common and still unknown risk factors, or if a synergic role of the two viruses is present, deserves further investigations.

## Conclusion

This study identified the presence of PCV-2 in a significant number of Namibian dogs, demonstrating the host plasticity of PCV-2 and the susceptibility of canines to the virus. The ingestion of contaminated pig meat or by-products, either produced locally or imported, is the most likely source of infection, although dedicated case–control or cohort studies should be performed to confirm this hypothesis. The epidemiological and clinical consequences are still unclear and further investigations are necessary. Nevertheless, the high proportion of infected dogs showing clinical signs raises concern about PCV-2’s role as a relevant viral pathogen in Namibia. The use of raw meat for dog nutrition should be discouraged, considering the known risks to animal and human health through disease transmission [30–33].

## Data Availability

All generated sequences have been made available in GenBank.
